# Assembly of a heterodinuclear Mn/Fe cofactor is coupled to tyrosine–valine ether cross-link formation in the R2-like ligand-binding oxidase

**DOI:** 10.1007/s00775-019-01639-4

**Published:** 2019-01-28

**Authors:** Julia J. Griese, Ramona Kositzki, Michael Haumann, Martin Högbom

**Affiliations:** 1 Department of Biochemistry and Biophysics, Stockholm University, 106 91 Stockholm, Sweden; 2 Department of Cell and Molecular Biology, Uppsala University, 751 24 Uppsala, Sweden; 3 Institut für Experimentalphysik, Freie Universität Berlin, 14195 Berlin, Germany

**Keywords:** Di-metal carboxylate protein, Ferritin, Ribonucleotide reductase, R2-like ligand-binding oxidase, X-ray crystallography

## Abstract

R2-like ligand-binding oxidases (R2lox) assemble a heterodinuclear Mn/Fe cofactor which performs reductive dioxygen (O_2_) activation, catalyzes formation of a tyrosine–valine ether cross-link in the protein scaffold, and binds a fatty acid in a putative substrate channel. We have previously shown that the N-terminal metal binding site 1 is unspecific for manganese or iron in the absence of O_2_, but prefers manganese in the presence of O_2_, whereas the C-terminal site 2 is specific for iron. Here, we analyze the effects of amino acid exchanges in the cofactor environment on cofactor assembly and metalation specificity using X-ray crystallography, X-ray absorption spectroscopy, and metal quantification. We find that exchange of either the cross-linking tyrosine or the valine, regardless of whether the mutation still allows cross-link formation or not, results in unspecific manganese or iron binding at site 1 both in the absence or presence of O_2_, while site 2 still prefers iron as in the wild-type. In contrast, a mutation that blocks binding of the fatty acid does not affect the metal specificity of either site under anoxic or aerobic conditions, and cross-link formation is still observed. All variants assemble a dinuclear trivalent metal cofactor in the aerobic resting state, independently of cross-link formation. These findings imply that the cross-link residues are required to achieve the preference for manganese in site 1 in the presence of O_2_. The metalation specificity, therefore, appears to be established during the redox reactions leading to cross-link formation.

**Electronic supplementary material:**

The online version of this article (10.1007/s00775-019-01639-4) contains supplementary material, which is available to authorized users.

## Introduction

Enzymes with a di-metal carboxylate cofactor catalyze numerous essential reactions and in particular reductive dioxygen (O_2_) activation in all kingdoms of life [1–5]. Three different combinations of manganese and iron have been found to constitute their cofactors. A diiron center is found in bacterial multicomponent monooxygenases (BMMs) as well as in the R2 subunit of class Ia ribonucleotide reductases (RNRs) [6–9]. In both groups the cofactor reduces O_2_, resulting in a high-valent metal–oxygen cofactor. In BMMs, this state catalyzes two-electron redox reactions, such as the hydroxylation of methane to methanol [7]. In contrast, in class Ia R2 proteins an electron from an exogenous donor is injected during O_2_ activation, resulting in a metal center that carries out a one-electron oxidation of a nearby tyrosine residue [6, 10]. The tyrosyl radical then initiates ribonucleotide reduction in the R1 subunit [6, 8, 9]. In class Ib RNRs, a dimanganese cofactor undergoes similar reactions, although it is activated by superoxide, which is provided by an additional flavodoxin subunit [11–17]. In the recently proposed class Id RNRs, which also bind a dimanganese cofactor, the radical equivalent appears to be stored at the metal site rather than at a tyrosine [18, 19]. Notably, a metal-free class Ie was also recently discovered [20–22]. The third metal combination identified to date is a heterodinuclear manganese/iron cofactor with the manganese ion occupying the N-terminal metal-binding site (site 1) [23–26]. This type of cofactor is found in class Ic R2 proteins (R2c), which store a radical equivalent on the cofactor after O_2_ reduction [27–29]. A related group of proteins with a similar Mn/Fe cofactor is termed R2-like ligand-binding oxidases (R2lox) [25, 26]. The physiological function of R2lox proteins has not yet been identified. Following O_2_ activation, their cofactor catalyzes a two-electron redox reaction which results in the formation of a tyrosine–valine ether cross-link in the protein [26, 30]. The tyrosine and valine residues are conserved in the R2lox group [4], and the cross-link was observed in both R2lox proteins that have been structurally characterized [25, 26]. Both proteins also comprise a hydrophobic channel leading to the metal cofactor wherein a long-chain fatty acid molecule is found, which coordinates at the Mn/Fe ions of the cofactor [25, 26].

The mixed-metal cofactor in R2lox self-assembles with the manganese ion in metal-binding site 1 and the iron ion in site 2 if both Mn^II^ and Fe^II^ are available [26]. Both a Mn/Fe and a Fe/Fe cofactor, but not a Mn/Mn or Fe/Mn cofactor, can be assembled in R2lox, and both cofactor types activate O_2_ and catalyze tyrosine–valine cross-link formation [30, 31], with the Mn/Fe cofactor being more efficient in this reaction [32]. How discrimination between manganese and iron in the two metal binding sites is achieved has remained unclear. Structural as well as kinetic reasons for metal discrimination have been discussed [26, 31, 33]. Our earlier studies of the wild-type R2lox protein showed that site 1 is unspecific for manganese or iron in the absence of O_2_, but prefers manganese in the presence of O_2_, indicating that metal specificity may be achieved not solely during the initial metal binding step, but at a later stage of the cofactor assembly pathway [26, 31]. Here we investigated the effect of amino acid point mutations on the metal specificity, redox state of Mn/Fe and Fe/Fe cofactors, and cross-link formation capability of R2lox using metal content quantification, X-ray absorption spectroscopy, and protein crystallography. We find that site 1 is only specific for manganese under aerobic conditions if the cross-link residues have their wild-type identity. It thus appears that cross-link formation and assembly of the heterodinuclear cofactor are synergistic.

## Materials and methods

### Site-directed mutagenesis, protein production and purification

Point mutations were introduced into a construct encoding full-length *Geobacillus kaustophilus* R2loxI (accession number WP_011232245) with an N-terminal hexahistidine tag in pET-46 Ek/LIC (Novagen) [26] by site-directed mutagenesis using the QuikChange Lightning kit (Agilent) and verified by DNA sequencing. The point mutations discussed in this study are V72A/I/L [32] and Y162F, the residues forming an ether cross-link in the wild-type protein, as well as A171F, a residue lining the ligand-binding pocket in the second coordination sphere of the metal cofactor. The R2lox variants were produced and purified in metal-free form as described previously [26]. Briefly, the protein was produced recombinantly in *E. coli* BL21(DE3) (Novagen) grown in terrific broth (ForMedium). To obtain metal-free protein, 0.5 mM EDTA was added to the cultures immediately before induction with 0.5 mM IPTG. Apo-protein was purified via heat denaturation of contaminating proteins and nickel chelate affinity chromatography. Cells were disrupted by high-pressure homogenization in lysis buffer (25 mM HEPES-Na, pH 7.0; 300 mM NaCl, 20 mM imidazole, 0.5 mM EDTA). The lysate was cleared by centrifugation, incubated at 333 K for 10 min, and again cleared by centrifugation. The supernatant was applied to a Ni–NTA agarose (Protino, Macherey-Nagel) gravity flow column. The beads were washed with lysis buffer containing 40 mM imidazole, followed by the same buffer lacking EDTA. Protein was then eluted using lysis buffer without EDTA containing 250 mM imidazole. The eluate was exchanged into storage buffer (25 mM HEPES-Na, pH 7.0; 50 mM NaCl) using a HiTrap Desalting column (GE Healthcare). The protein was concentrated to approximately 1 mM, aliquoted, flash-frozen in liquid nitrogen and stored at 193 K [26]. The protein concentration was measured using experimentally determined extinction coefficients at 280 nm [34]. The extinction coefficients are 47.8 mM^−1^ cm^−1^ or 50.6 mM^−1^ cm^−1^ for metal-free or metal-bound wild-type R2lox [34] and all point mutants except Y162F-R2lox, and 43.9 mM^−1^ cm^−1^ or 46.6 mM^−1^ cm^−1^ for metal-free or metal-bound Y162F-R2lox, respectively. The metal contents of purified apo-protein batches were quantified by TXRF (see below).

### Crystallization and data collection

All R2lox variants were crystallized in metal-free form by vapor diffusion in hanging drops at 295 K. Wild-type, A171F- and V72I-R2lox crystallized in 25–30% (w/v) PEG 1500, 100 mM HEPES-Na, pH 7.4–7.5. Only intergrown crystals containing multiple lattices could be obtained of Y162F-R2lox in the wild-type condition as well as upon re-screening, whereas the V72A and V72L mutants did not nucleate at all. To obtain single crystals of these three mutants, drops were, therefore, streak-seeded with wild-type R2lox crystals following a 1 h equilibration after setting up the hanging-drop plates, with the mother liquor containing 20–22.5% (w/v) PEG 1500 and 100 mM HEPES-Na, pH 6.8–7.0. Crystals grew in clusters along the streak line, but could easily be separated into large three-dimensional single crystals. To reconstitute the oxidized resting state Mn/Fe cofactor in R2lox point mutants, crystals of metal-free protein were removed from their drop and soaked in mother liquor additionally containing 5 mM each MnCl_2_ and (NH_4_)_2_Fe(SO_4_)_2_ for 1–2 h under aerobic conditions and then briefly washed in 40% (w/v) PEG 1500, 100 mM HEPES-Na (at the pH of the mother liquor) before flash-cooling in liquid nitrogen. To obtain the non-activated reduced Mn/Fe cofactor, apo-protein crystals were soaked in 1 ml of 40% (w/v) PEG 1500, 100 mM HEPES-Na (at the pH of the mother liquor), 5 mM (NH_4_)_2_Fe(SO_4_)_2_, 5 mM MnCl_2_, 0.5% (w/v) sodium dithionite, 0.5 mM phenosafranin, and 0.05% (v/v) Tween 20 for 1–2 h and flash-cooled directly without washing [26]. Soaking solutions were freshly prepared immediately before use, using freshly dissolved (NH_4_)_2_Fe(SO_4_)_2_ and dithionite to ensure that the Fe was ferrous, and that O_2_ was effectively removed from soaking solutions used to obtain reduced states, with phenosafranin serving as a redox indicator. We note that the soaking duration has been shown to have no effect on final active site metal contents [26]. Diffraction data were collected at 100 K at beamlines X06SA and X10SA at the Swiss Light Source (SLS, Villigen, Switzerland) and BL14.1 at BESSY (Helmholtz Center Berlin, Germany). For the purpose of metal quantification, data collection proceeded in the order Fe K-edge—Mn K-edge on the same crystal. High-resolution data were collected after anomalous datasets.

### Structure determination, model building, and refinement

Data were processed with XDS [35]. The structures of R2lox point mutants were solved using the structure of the wild-type protein in the same redox state [26] not containing any ligands as a starting model. Crystals of A171F-R2lox were obtained in the same space group (I222) as the wild-type with one molecule in the asymmetric unit, and these structures were consequently solved by Fourier synthesis. Crystals of the Y162F mutant were in space group C2 with two molecules in the asymmetric unit. These structures were, therefore, solved by molecular replacement using Phaser in Phenix [36, 37]. Refinement was carried out with phenix.refine [36, 38] and iterated with rebuilding in Coot [39]. Refinement generally included bulk solvent corrections, individual atomic coordinate and isotropic *B* factor refinement, and occupancy refinement for alternate conformations and metal ions bound on the protein surface, but not the active site metal ions. Metal–ligand bond lengths were restrained. Solvent molecules were added with phenix.refine as well as manually. Hydrogens were added to the models in the later stages of refinement. One exception was made to this general protocol: In the structure of reduced state Y162F-R2lox, anisotropic *B* factors were refined due to the sufficiently high resolution of the data (1.4 Å). Structures were validated using MolProbity [40]. Data and refinement statistics are given in Tables S1 and S2. Figures were prepared with PyMOL (version 1.8.6.2, Schrodinger, LLC).

### Analysis of anomalous diffraction data

All anomalous datasets were integrated over the same resolution range (50.0–3.0 Å) with XDS [35] (Table S1). The Fe and Mn edge datasets from one crystal were placed on a common scale with XSCALE [35]. Both scaled and unscaled datasets were analyzed. Anomalous difference maps were calculated with PHENIX [36] using the phases from a ligand-free model, and the relative amounts of Fe and Mn in each metal site were calculated as previously described [26]. The intensities of the anomalous difference density peaks in spheres of 1.9 Å radius around the center of the peaks were integrated using Mapman [41]. The relative amounts of Fe and Mn in each site were estimated from the integrated intensities at the Fe and Mn edges by taking into account the different contributions of both elements to the anomalous signal at the two wavelengths, assuming a total occupancy of each site of 1. Since only relative amounts are calculated, the actual occupancy is irrelevant as long as it is high enough to yield a significant anomalous peak, which was the case in all datasets. The quantification results for scaled and unscaled datasets matched within 10%.

### X-ray absorption spectroscopy (XAS)

To reconstitute oxidized resting-state Mn/Fe and Fe/Fe cofactors in the V72A-, Y162F- and A171F-R2lox variants as well as wt-R2lox for XAS analysis, 250 µM apo-protein (monomer concentration) was incubated with 2 equivalents (per monomer) of MnCl_2_ and 1 equivalent of (NH_4_)_2_Fe(SO_4_)_2_ or 3 equivalents of (NH_4_)_2_Fe(SO_4_)_2_ only in reconstitution buffer (100 mM HEPES-Na, pH 7.0; 50 mM NaCl) for 1 h at room temperature under aerobic conditions. Excess metal ions were removed by passing the samples through a HiTrap Desalting column (GE Healthcare) equilibrated in storage buffer. The reconstituted protein was concentrated to 1–3.5 mM, transferred into sample holders and frozen in liquid nitrogen. XAS spectra at the Mn and Fe K-edges were collected at the SuperXAS beamline of SLS (Villigen, Switzerland) using a fluorescence-detection set-up (Si[111] double-crystal monochromator, 5-element silicon-drift detector, samples held in a liquid-He cryostat from Cryovac at 20 K) and previously described procedures for data collection, evaluation, and processing [30]. A linear fit to the pre-K-edge region was subtracted from the XAS spectra for background removal. The resulting XAS spectra were normalized (to an edge jump of unity) by division by a 3rd-order polynomial through the post-K-edge region. X-ray absorption near edge structure (XANES) spectra were normalized after averaging of up to 15 scans of approximately 2 min duration on separate sample spots (using appropriate beam attenuation to avoid X-ray photoreduction of cofactors), detector dead-time correction, and energy axis calibration. The monochromator energy axis was calibrated using the first inflection point at 7112.0 eV in the 1st derivative of the absorption spectrum of an iron foil (Fe K-edge) or the pre-edge peak maximum at 6543.3 eV in the absorption spectrum of a permanganate (KMnO_4_) powder sample (Mn K-edge) as reference energies [42, 43].

### Total-reflection X-ray fluorescence (TXRF) analysis of protein metal contents

To assess the cofactor assembly mechanism in solution, R2lox point mutants were reconstituted using different ratios of protein:Mn:Fe in reconstitution buffer at room temperature under aerobic conditions. Samples were prepared in duplicate. Mn titration series were prepared by adding 2, 4 or 8 equivalents (per monomer) of MnCl_2_ to 100 µM apo-protein prior to addition of 2 equivalents (NH_4_)_2_Fe(SO_4_)_2_ and incubating the samples for 1 h. Fe titration series were prepared by adding 4 equivalents of MnCl_2_ to 100 µM apo-protein. Then, 0.2 equivalents of (NH_4_)_2_Fe(SO_4_)_2_ were added from a 10 mM stock solution (i.e. protein:Mn:Fe = 1:4:0.2). After 20 min incubation, an aliquot was taken, and another 0.4 equivalents of (NH_4_)_2_Fe(SO_4_)_2_ were added to the remaining solution (i.e. protein:Mn:Fe = 1:4:0.6). This step was repeated once more (i.e. protein:Mn:Fe = 1:4:1). All Fe titration samples were incubated for 1 h after the last addition of (NH_4_)_2_Fe(SO_4_)_2_, i.e. for a total of 100 min. Following the reconstitution procedure, excess metal ions were removed by passing the samples through a HiTrap Desalting column (GE Healthcare) equilibrated in storage buffer. The samples were concentrated to approximately 0.5 mM protein, which was determined as above. Metal contents of apo-protein preparations and metal titration samples were quantified using TXRF analysis on a Bruker PicoFox instrument [44]. A gallium standard (Sigma) was added to the samples (v/v 1:1) prior to the measurements. TXRF spectra were analyzed using the routines provided with the spectrometer.

## Results

Five different amino acid point mutations were introduced in the R2lox scaffold, and their effects on the structure and metal specificity of the cofactor were investigated. Four mutations were made to the cross-linking residues, which are conserved in the R2lox group [4]. The valine was changed to alanine, isoleucine, or leucine (V72A/I/L) [32], and the tyrosine was changed to phenylalanine (Y162F). Moreover, A171, which lines the fatty-acid binding tunnel, was changed to phenylalanine (A171F). The latter mutation was designed to create a cofactor geometry in R2lox more closely resembling the R2c site by blocking access of the fatty acid ligand that co-purifies with the wild-type protein and coordinates both metal ions. The A171 position is conserved as an alanine in the R2lox family, whereas in the corresponding position a phenylalanine is conserved among all groups of RNR R2 proteins [4].

### Crystal structures of R2lox variants

**Fig. 1:**
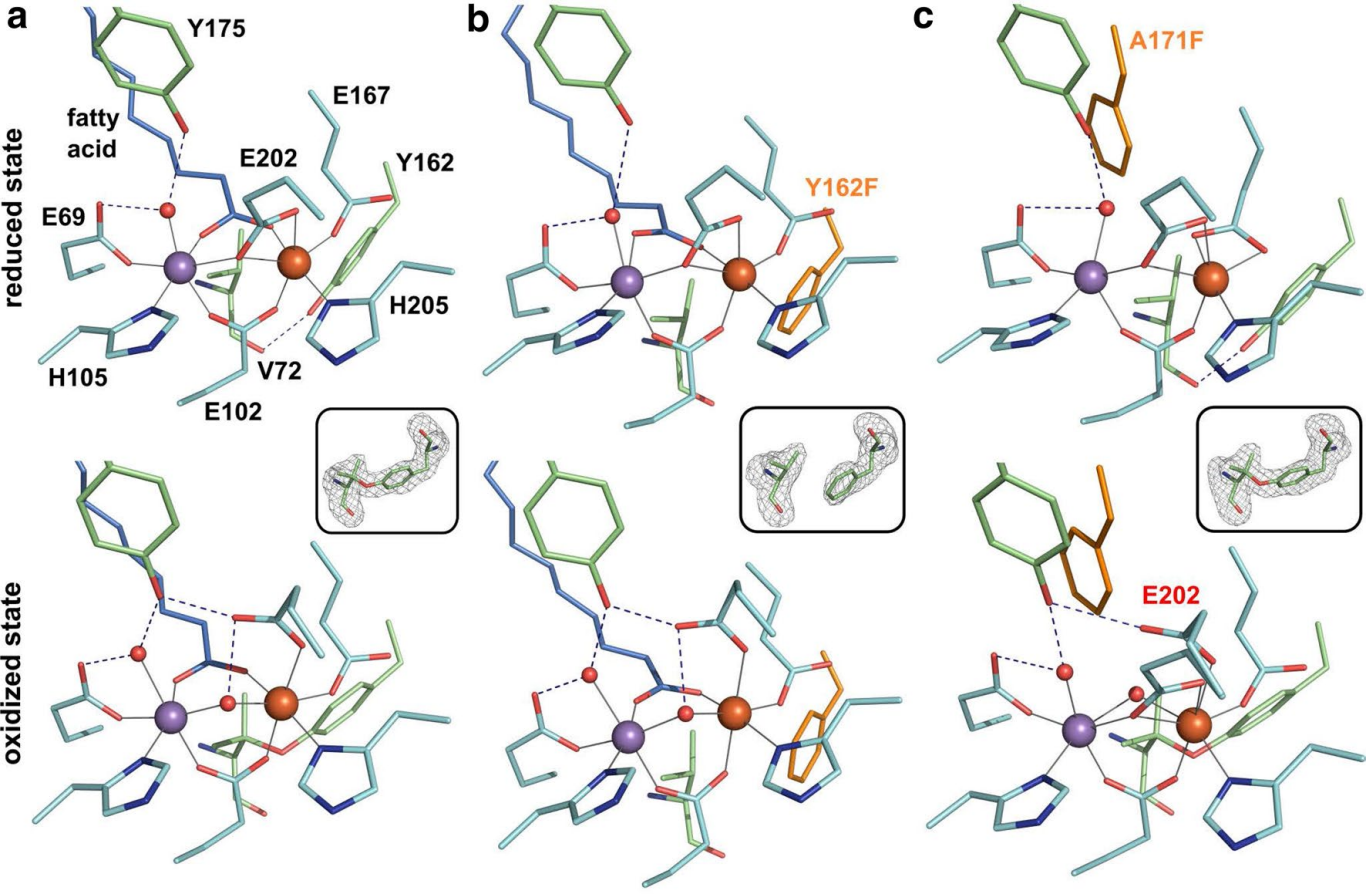
Active site structures of R2lox variants in the non-activated reduced (top panel) and O_2_-oxidized resting state (bottom panel). All structures are shown in roughly the same orientation, with site 1 on the left. Mutated residues are highlighted in orange. Metal–ligand bonds are indicated by grey lines, hydrogen bonds by dashed blue lines. The insets show *mF*_*o*_–*DF*_*c*_ refined omit electron density contoured at 3.0*σ* for residues 72 and 162 in oxidized state crystals. **a** wt-R2lox (reduced state, PDB ID 4HR4; oxidized state, PDB ID 4HR0) [26]: an ether cross-link is formed between the Cβ of V72 and the hydroxyl oxygen of Y162 in the oxidized state. **b** Y162F-R2lox: no ether cross-link is formed. **c** A171F-R2lox: the V72-Y162 ether cross-link is formed. In the oxidized state, E202 (labeled in red) is observed in two alternate conformations, one leading to a hexacoordinate Fe ion, the other leaving one coordination site vacant, unless a second oxo/hydroxo bridge (not modeled) takes its place (the metal–ligand bonds of both conformations are indicated)

The crystal structures of wt-R2lox and the V72 mutants in the non-activated reduced and O_2_ oxidized resting state have previously been described [26, 30, 32], but will briefly be discussed here for comparison. In wt-R2lox, a manganese and an iron ion are bound next to each other in octahedral geometry by two histidine and four glutamate residues [26, 30] (Fig. 1a). In addition to the amino acid ligands, a long-chain fatty acid ligand bridges the metal ions and a water molecule is bound at the manganese ion in site 1. After oxidation of the cofactor by O_2_, a µ-hydroxo bridge displaces the C-terminal glutamate ligand from its bridging/chelating position, and an ether cross-link is formed between the Cβ of V72 and the phenolic oxygen of Y162 [26, 30, 33, 34]. Prior to cross-link formation, the hydroxyl group of Y162 is hydrogen-bonded to the carbonyl oxygen of V72 [26, 30]. Mutations of V72 have little effect on the global structures of the reduced and oxidized resting cofactor states and cause only minor changes to the metal coordination geometry. Importantly, none of the mutations appears to inhibit O_2_ reduction and metal oxidation, but mutation of V72 to alanine or leucine prevents cross-link formation, whereas tyrosine–isoleucine cross-link formation is observed with an isoleucine in place of the native valine [32].

The mutation of the cross-link tyrosine to a phenylalanine was found to have little effect on the active site and global protein structure of R2lox, although the hydrogen bond to the carbonyl oxygen of V72 is missing in the reduced state and the cross-link cannot be formed upon oxidation (Fig. 1b). Interestingly, in the structure of the aerobically reconstituted protein, only one of the two subunits of the dimer in the asymmetric unit of the crystals is in the oxidized state conformation. Very weak difference density for the oxidized conformation in the other subunit suggested that the reduced state conformation was present in at least 90% of this molecule (not shown). This asymmetric behavior might be caused by different packing interactions of the two molecules in the crystal. It should be noted, however, that other mutants that crystallized in the same space group (V72A/L-R2lox) did not display large differences between the two subunits [32]. It appears, therefore, that the asymmetry is caused by the Y162F mutation, either because O_2_ activation is partially impaired in Y162F-R2lox, or because one subunit is more sensitive to X-ray photoreduction during diffraction data collection, an effect previously observed in a related class Ib R2 protein [45].

In A171F-R2lox, the fatty acid is excluded from the active site because the bulky phenylalanine side chain blocks the ligand-binding tunnel (Fig. 1c). Electron density for a hydrophobic ligand is observed further up in the tunnel, but it could only reasonably be modeled (as caprylic (C_8_) acid) in the oxidized state. Similar to the V72L variant [32], in the reduced state of A171F-R2lox, E167 ligates Fe2 in bidentate mode instead of the monodentate ligation in the wild-type, so that Fe2 adopts a near-octahedral geometry as in the presence of the fatty acid ligand, while Mn1 appears to have an unoccupied coordination site. The other metal ligands retain the same spatial configuration as in the wild-type. In contrast, in the oxidized state of A171F-R2lox, E167 is a monodentate ligand to Fe2 as in the wild-type, whereas E202 adopts the oxidized or reduced state conformation in roughly equal proportions. However, cofactor oxidation by O_2_ appears to have taken place regardless of the E202 conformation, because an oxo/hydroxo bridge is clearly observed in the electron density in the position occupied by the fatty acid head group in the wild-type. It appears likely that in those molecules in which E202 is in the oxidized state conformation, a second oxo/hydroxo species occupies the bridging position. Accordingly, the A171F-R2lox cofactor can adopt a geometry which is similar to the oxidized cofactor in R2c [29], with two oxygen bridges instead of only one as in wt-R2lox. However, none of these structural changes seem to have an effect on cofactor reactivity with respect to cross-link formation. Judging from the electron density, the cross-link was formed equally efficiently in the presence of O_2_ in A171F-R2lox as in the wild-type protein (Fig. 1c, inset).

### Metal oxidation states in aerobically reconstituted R2lox variants

**Fig. 2:**
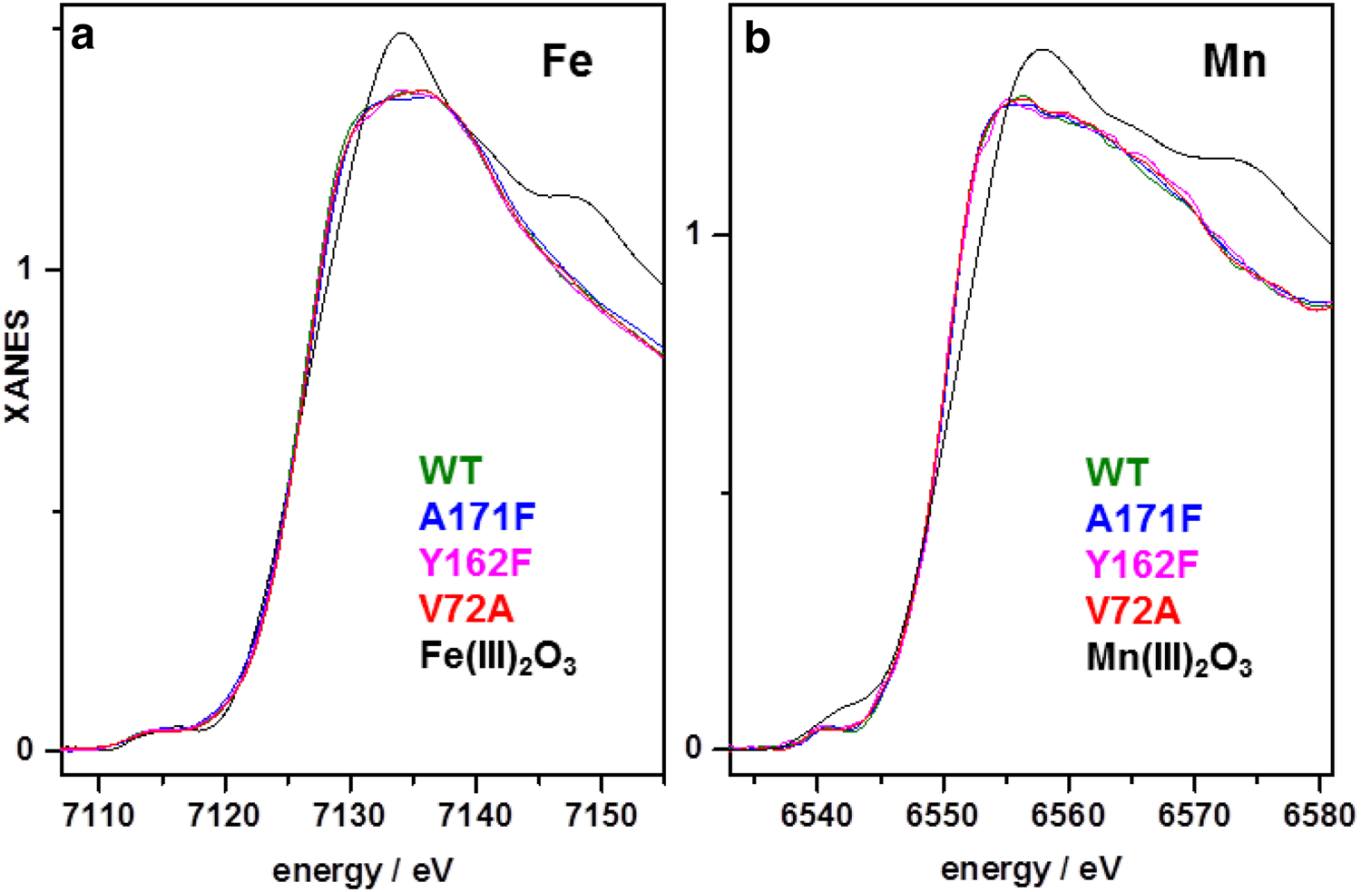
X-ray absorption spectra of aerobically metal-reconstituted wt-, V72A-, Y162F- and A171F-R2lox variants. **a** Fe K-edge spectra of Fe-only reconstituted variants; **b** Mn K-edge spectra of Mn/Fe-reconstituted variants. The spectra of solid Fe^III^ or Mn^III^ oxides are shown for comparison

X-ray absorption spectroscopy was carried out on selected R2lox variants to study the metal oxidation state (Fig. 2). The Mn K-edge spectra of Mn/Fe-reconstituted samples and the Fe K-edge spectra of Fe/Fe-reconstituted wt-, V72A-, Y162F-, and A171F-R2lox prepared under aerobic conditions were very similar, confirming the overall similar, near-octahedral metal coordination observed in the crystals. The almost identical K-edge energies (Fe, 7124.8 ± 0.1 eV; Mn, 6549.2 ± 0.1 eV; at 50% edge-rise level) of all variants were similar to our previously determined K-edge energies (Fe, 7124.8 ± 0.1 eV; Mn, 6549.3 ± 0.1 eV) of the oxidized wild-type protein [30], indicating the near-quantitative presence of Mn^III^ and Fe^III^. Accordingly, all R2lox variants assemble (Mn/Fe)^III^/Fe^III^ cofactors in the presence of O_2_, independently of the metal ligation motif and ability to form the cross-link. No impairment of O_2_ reduction could be observed in Y162F-R2lox in solution. This suggests that enhanced sensitivity to photoreduction was the reason for the reduced state conformation that is prevalent in one of the two subunits of the oxidized state Y162F-R2lox crystal structure (see above).

### Metal distribution in the active site of R2lox variants following crystal soaking

**Fig. 3:**
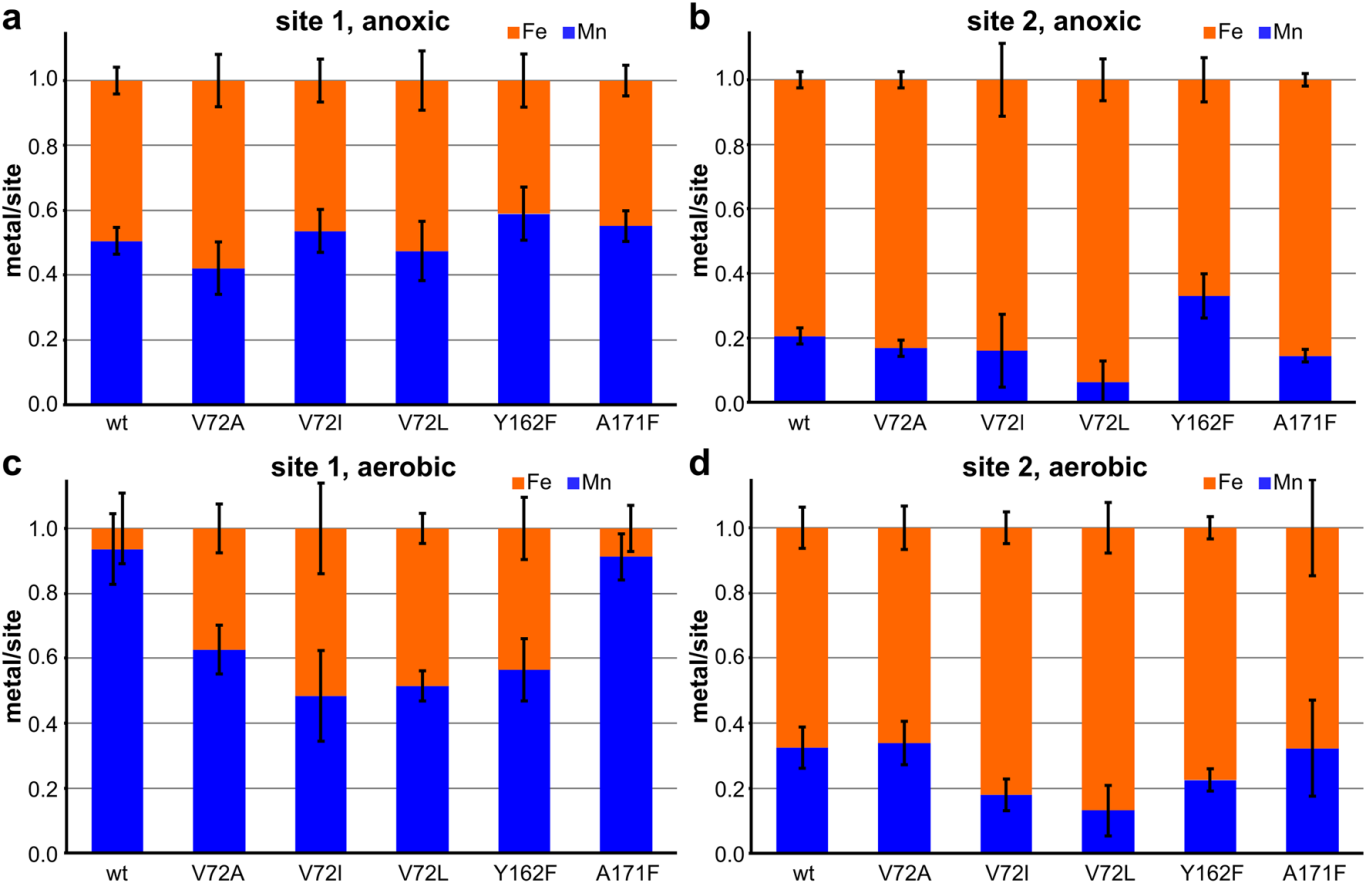
Relative amounts of Mn and Fe in metal sites 1 (**a**, **c**) and 2 (**b**, **d**) of R2lox variants following crystal soaking with Mn^II^ and Fe^II^ in the absence (**a**, **b**) or in the presence (**c**, **d**) of O_2_, compared to wt-R2lox [26]. The column height of 1 refers to the sum of Mn and Fe in each metal site. Metal-free protein crystals were soaked with an excess of Mn^II^ and Fe^II^ in equal concentrations under reducing anoxic (0.5% sodium dithionite) or aerobic conditions (air-saturated buffer). Relative metal amounts are derived from the integrated intensity of the anomalous difference density peaks at the Mn and Fe absorption edges. Data stem from at least 2 crystals in space group C2 with two molecules per asymmetric unit, or at least 4 crystals in space group I222 with one molecule per asymmetric unit, i.e. at least 4 independent observations of each site (see Table S1). Error bars show standard deviations of the replicates

To investigate whether the point mutations have an effect on metal specificity, we soaked metal-free protein crystals with an excess of both metal(II) ions in equal concentrations under anoxic and aerobic conditions and analyzed the relative amounts of Mn and Fe in each metal-binding site using X-ray anomalous dispersion, as previously done with the wild-type protein [26] (Fig. 3). In wt-R2lox, roughly 1:1 Mn:Fe accumulate in site 1 and mainly Fe in site 2 under anoxic conditions, whereas under aerobic conditions site 2 also contains mainly Fe, but site 1 contains almost exclusively Mn [26]. The same metal distribution was observed in A171F-R2lox. In stark contrast, in all four cross-link residue mutants, site 1 contained roughly 1:1 Mn:Fe under anoxic as well as under aerobic conditions, while the metal distribution in site 2 was about the same as in the wild-type. In V72A/L- and Y162F-R2lox, both subunits of the dimer in the asymmetric unit had the same metal distribution. It should be noted that the difference between the metal contents in wt- and A171F-R2lox on the one hand and the cross-link mutants on the other hand cannot be attributed to the different crystallographic space groups these R2lox variants adopt, nor to the different pH values of the crystallization mother liquor. While the V72A/L and Y162F mutants crystallized in a different space group and at a lower pH than the wild-type (and required seeding with wild-type crystals), V72I-R2lox crystallized in the same space group and at the same pH as wt-R2lox (see Materials and Methods and Supporting Information, Table S1). It should also be noted that the V72A/L- and Y162F variants do not form the ether cross-link, while the V72I variant does, but the metal distribution is virtually identical in all these variants and unlike that of the wild-type.

### Metal contents of R2lox variants after reconstitution in aerobic solution

**Fig. 4:**
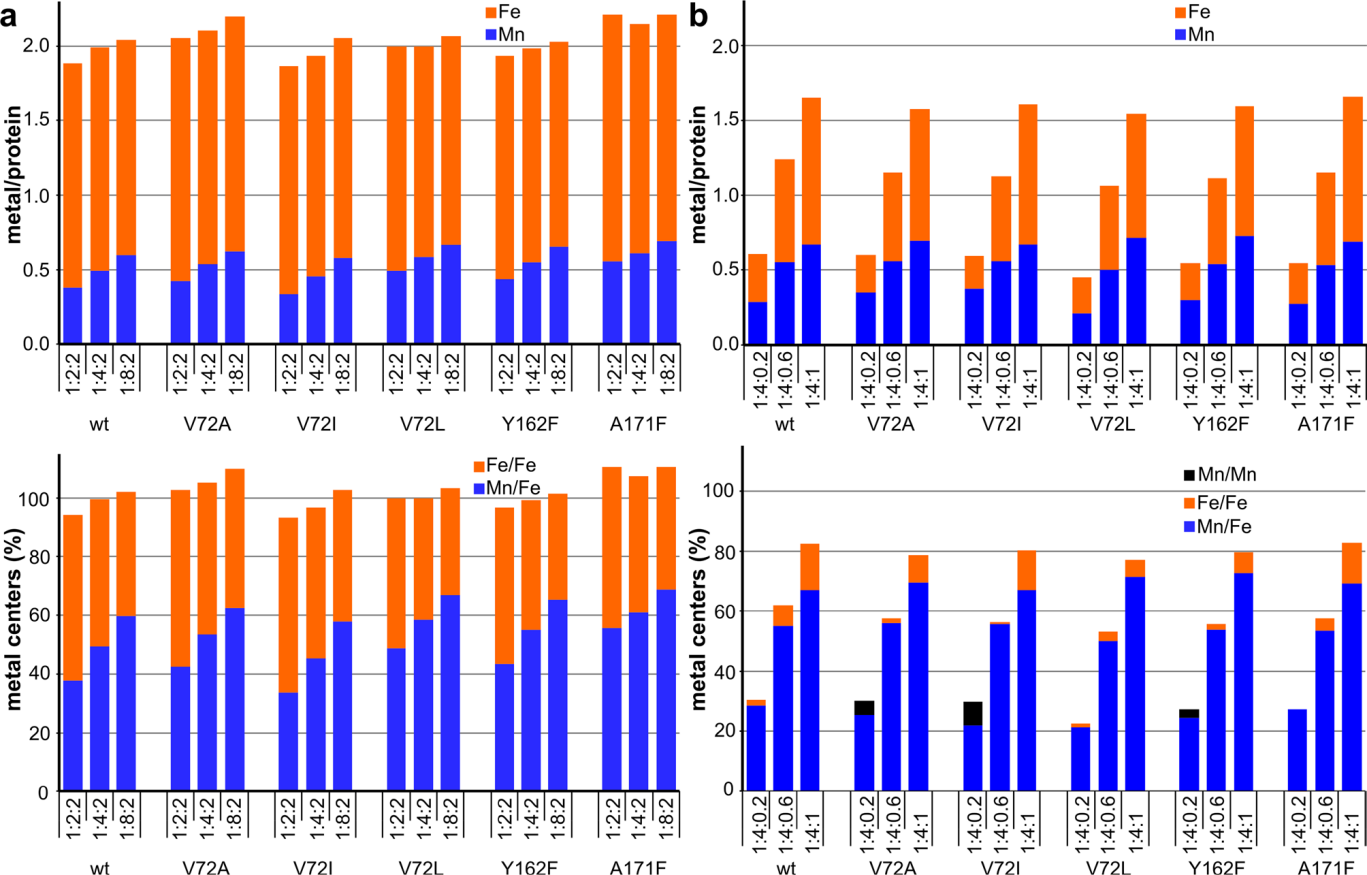
Mn and Fe contents of R2lox variants after reconstitution with Mn^II^ and Fe^II^ in solution, as determined by TXRF. **a** Mn titration; **b** Fe titration. *X* axis labels denote protein:Mn:Fe ratios in the cofactor reconstitution. Column heights show the sum of Mn and Fe per protein in the top panels and of Mn/Fe, Fe/Fe and Mn/Mn cofactor content in the bottom panels. Shown are mean values from two independent measurements each on duplicate samples. The error is less than or equal to 0.1 metal ions per protein. Other transition metals (Ni, Cu, Zn) were negligible in all samples. Proportions of metal center species in the bottom panel were calculated assuming that all metal ions are bound in dinuclear centers and that formation of heterodinuclear clusters is preferred

We also investigated the metal binding behavior of the R2lox variants in solution for comparison with the crystallographic results. Only up to 0.6 Mn/protein are accumulated in wt-R2lox in solution when Mn is in excess and Fe is substoichiometric, whereas stoichiometric amounts of Mn and Fe lead to more Fe/Fe than Mn/Fe centers being formed [31]. Here, we reconstituted wt-R2lox and the five variants with Mn^II^ and Fe^II^ at different protein:Mn:Fe ratios in aerobic solution and analyzed the metal contents of the proteins by TXRF after removal of excess metal ions. The apo-protein preparations were also analyzed and found to contain no more than 0.1 Fe/protein and no significant amounts of other transition metals. As suggested by previous analyses [31], cofactor distributions were calculated assuming that all metal is bound in dinuclear centers and that stoichiometric manganese is incorporated in Mn/Fe sites (with Mn in site 1 as observed in crystals). In contrast to the crystallographic experiments, in solution none of the mutations had a significant effect on the metal contents of the R2lox variants compared to the wild-type (Fig. 4). Reconstitution with 2 equivalents of Fe and 2, 4, or 8 equivalents of Mn led to significant amounts of diiron clusters, and a maximum of approximately 60–70% of the protein contained Mn/Fe cofactors, in agreement with our earlier results [31]. When up to 1 equivalent Fe was slowly titrated in after addition of 4 equivalents Mn to the protein, a maximum of 0.7 Mn/Fe clusters per protein was obtained, but under these conditions the remaining protein was largely metal free, with only approximately 10% occupied by diiron centers, again in agreement with earlier data [31]. The data suggest that, in contrast to the wild-type, V72A/I- and Y162F-R2lox accumulated a small amount of Mn/Mn cofactors when only 0.2 equivalents of Fe had been added. However, the amount of Mn per protein which was surplus to the amount of Fe in these samples is within the error range of the quantification. Under the present solution conditions, all studied R2lox variants, therefore, showed similar metal binding site occupation as the wild-type.

## Discussion

We show here that the cross-link residues are required for efficient assembly of the Mn/Fe cofactor in R2lox. Unlike the mutation of A171 in the fatty-acid binding tunnel to phenylalanine, a mutation in either of the cross-link residues, even if it does not prevent cross-link formation, abolishes the manganese specificity of site 1 observed in the wild-type protein. Together with the results from the metal reconstitution procedure variations, the mutational data provide further insight into the cofactor assembly pathway of R2lox.

R2lox displays different metal preferences in the absence and presence of O_2_, indicating that site-selective metal specificity is determined by a combination of the protein scaffold’s intrinsic metal(II) preferences and differential preferences for metal(III) ions established during O_2_ binding and/or reduction. In the wild-type protein, site 2 always prefers iron, while site 1 is unspecific for Mn^II^ or Fe^II^ under anoxic conditions, but strongly prefers Mn^III^ over Fe^III^ under aerobic conditions [26]. We have previously proposed that manganese is preferred in site 1 because the presence of a terminal water ligand pre-adapts this site for accommodation of a Mn^III^ (pseudo-) Jahn–Teller ion with its axis along the Mn–water bond, whereas the octahedral site 2, which contains only terminal amino acid ligands, is more suitable for iron binding [31, 33].

The preference for manganese in site 1 is more clearly observed in the crystal soaking experiments compared to solution conditions [26, 31], suggesting that the ratio of available metal ions to protein plays an important role in cofactor assembly. In the crystal soaking experiments, metal ions were in large excess and, taking into account the low O_2_ concentration (approximately 0.25 mM in O_2_-saturated buffer at room temperature), metal exchange may be faster than O_2_ reduction under these conditions [46], so that the preferred cofactor configuration can be formed before O_2_ activation is completed, and the relative Mn/Fe cofactor content is thereby increased. In solution, protein precipitation prevents the use of a large excess of metal ions. Under these conditions, with near-stoichiometric metal(II) to protein ratios, any initially formed, catalytically competent cofactor, i.e. Mn^II^/Fe^II^ or Fe^II^/Fe^II^ [30, 31], is stabilized in a higher-valent state because metal exchange is likely considerably slower than O_2_ reduction [46], so that the final metal content of the protein is determined primarily by the metal(II) preferences of the sites, similarly to the anoxic crystal soaks. The results shown here demonstrate that the studied second-sphere mutations do not influence the metal(II) preferences of R2lox, suggesting that these preferences are primarily determined by the structure of the first ligand sphere. However, the metal(II) affinity of R2lox is generally low, whereas under aerobic conditions dinuclear metal(III) cofactors are efficiently stabilized [31, 46], presumably because the stronger metal–ligand bonds and the metal-bridging oxide stabilize the metal(III)-containing cofactors.

The R2lox variants with exchanged cross-link residues show that the native cross-link residues are required to achieve the manganese preference of site 1 under aerobic conditions. The different metal preferences of the cross-link mutants in comparison to the wild-type protein are not caused by a lack of O_2_ reduction and metal oxidation, as the crystal structures and the XAS data demonstrate that all variants activate oxygen [32]. Like the wild-type [30, 33], the Y162F and V72A mutants assemble mostly (Mn/Fe)^III^/Fe^III^ cofactors, suggesting that in the absence of cross-link formation, putative metal(IV) species formed after full O_2_ reduction are partially reduced by external electron sources. Such unspecific reduction, as well as metal reduction in the course of cross-link formation in V72I-R2lox, however, presumably occur at a different rate than reduction by cross-link formation in the wild-type. We, therefore, propose that the cross-link residues and the actual cross-link formation in wt-R2lox control the decay rate of initial high-valent states of the cofactor, which, in relation to the rate of metal exchange, leads to the preferential assembly of Mn^III^/Fe^III^ cofactors. We note that this would require that some steps of the oxygen reduction mechanism are reversible. In support of this hypothesis, we have recently shown that the Mn/Fe cofactor catalyzes cross-link formation more efficiently than its diiron counterpart in wt-R2lox [32]. Taken together, these results indicate that assembly of the heterodinuclear Mn/Fe cofactor is coupled to cross-link formation.

Cofactor assembly in R2lox thus appears to be controlled by a combination of thermodynamic and kinetic effects and to be highly dependent on the relative rates of Mn^II^, Fe^II^ and O_2_ access, prompting the question whether this mechanism is relevant in vivo. While total metal concentrations in the cell are in the mM range, similar to the crystal soaking conditions, the amount of free metal is expected to be very low [47], more similar to the solution conditions described here. Which amounts of metal ions are accessible to the protein at which rates in competition with the numerous other chelators in the cell, as well as the rate of O_2_ supply, is a difficult problem to approach experimentally and thus also to reproduce in vitro. Moreover, expression in heterologous hosts and/or overexpression, in general, will also result in conditions for cofactor assembly that differ from those in the native host at native expression levels. This challenge is principally encountered when addressing metal cofactor assembly mechanisms for all metalloproteins for which no specific metallochaperone-mediated assembly pathway is present. Most likely, this concerns the vast majority of Fe and Mn-cofactor proteins.

Here, we describe two different in vitro conditions for cofactor assembly, in solution with low metal concentration and in crystals with high metal concentration. While the first may intuitively appear more similar to the in vivo situation, we note that when R2lox is produced aerobically in vivo without added metal chelators, the resulting protein contains predominantly heterodinuclear Mn/Fe cofactors [25]. Thus, the crystal soaking experiments, in fact, appear to better reflect the in vivo outcome than the solution reconstitution experiments. In summary, these data imply that the biologically relevant form of R2lox contains both a Mn/Fe cofactor and the tyrosine–valine cross-link.

## Electronic supplementary material

Below is the link to the electronic supplementary material.

Supplementary material 1 (PDF 130 kb)

## Abbreviations

BMM: Bacterial multicomponent monooxygenase
RNR: Ribonucleotide reductase
R2c: Class Ic RNR R2 protein
R2lox: R2-like ligand-binding oxidase
TXRF: Total-reflection X-ray fluorescence
wt: Wild-type
XANES: X-ray absorption near edge structure
XAS: X-ray absorption spectroscopy
