# Supplementary material for: Assembly of a heterodinuclear Mn/Fe cofactor is coupled to tyrosine–valine ether cross-link formation in the R2-like ligand-binding oxidase
Source: J Biol Inorg Chem. 2019 Jan 28;24(2):211–21. doi: 10.1007/s00775-019-01639-4 (PMC6399176; doi:10.1007/s00775-019-01639-4)
Supplement: Supplementary file 1 — Supplementary material 1 (PDF 130 kb) [file 775_2019_1639_MOESM1_ESM.pdf]

Electronic Supplementary Material for:

Assembly of a heterodinuclear Mn/Fe cofactor is coupled to tyrosine-valine ether cross-link formation in the R2-like ligand-binding oxidase

Julia J. Griese[1,2](✉), Ramona Kositzki[3], Michael Haumann[3], and Martin Högbom[1](✉)

[1]Department of Biochemistry and Biophysics, Stockholm University, SE-106 91 Stockholm, Sweden; [2]Department of Cell and Molecular Biology, Uppsala University, SE-751 24 Uppsala, Sweden; [3]Institut für Experimentalphysik, Freie Universität Berlin, D-14195 Berlin, Germany.
✉ E-mail: julia.griese@icm.uu.se; hogbom@dbb.su.se

The Electronic Supplementary Material contains:
Table S1 Crystallographic data statistics.
Table S2 Refinement statistics.
References

Table S1 Crystallographic data statistics

**R2lox variant**	**V72A**					
**Soaking condition**	**anoxic Mn+Fe**					
Crystal	1		2		3	
Beamline	X06SA/SLS	X06SA/SLS	X06SA/SLS	X06SA/SLS	X06SA/SLS	X06SA/SLS
Detector	Pilatus 6MF	Pilatus 6MF	Pilatus 6MF	Pilatus 6MF	Pilatus 6MF	Pilatus 6MF
Dataset	Fe peak	Mn peak	Fe peak	Mn peak	Fe peak	Mn peak
Wavelength (Å)	1.73	1.88	1.73	1.88	1.73	1.88
Resolution range (Å)	50.00-2.98 (3.16-2.98)	50.00-2.98 (3.16-2.98)	50.00-3.00 (3.18-3.00)	50.00-2.97 (3.15-2.97)	50.00-3.00 (3.18-3.00)	50.00-2.99 (3.17-2.99)
Space group	C2	C2	C2	C2	C2	C2
Unit cell dimensions a, b, c (Å)	161.36, 55.62, 70.04	161.48, 55.63, 70.07	161.77, 55.71, 70.18	161.70, 55.69, 70.13	162.13, 55.58, 70.10	162.13, 55.59, 70.12
β (°)	114.13	114.13	114.18	114.18	114.34	114.35
Unique reflections	21331 (3206)	20921(2970)	21837 (3422)	21525 (2892)	21869 (3447)	21697 (3384)
Multiplicity	3.4	3.3	3.4	3.3	3.5	3.4
Completeness (%)	93.8 (86.8)	91.5 (81.3)	96.9 (93.3)	93.1 (77.9)	97.1 (94.3)	96.1(92.4)
I/σ(I)	25.91 (12.73)	16.04 (6.38)	24.88 (12.25)	12.91 (4.92)	20.20 (10.82)	14.94 (6.52)
R_{merge} (%)	3.7 (8.0)	5.7 (14.5)	3.8 (8.4)	7.1 (19.0)	4.9 (9.4)	6.3 (15.0)
R_{meas} (%)	4.4 (9.6)	6.8 (17.6)	4.5 (10.1)	8.5 (23.1)	5.8 (11.6)	7.6 (18.1)
$CC_{1/2}$ [a]	99.8 (99.3)	99.6 (97.6)	99.8 (99.1)	99.5 (96.0)	99.7 (98.8)	99.6 (97.3)
Anomalous signal	1.37 (1.11)	0.97 (0.95)	1.35 (1.01)	0.87 (0.86)	1.16 (0.96)	0.87 (0.81)

Values in parentheses are for the highest resolution shell. For native datasets, Friedel pairs were merged [a]Percentage of correlation between intensities from random half-datasets [1]. The correlation is significant at the 0.1% level in all resolution shells in all datasets.

Table S1 (continued) Crystallographic data statistics

**R2lox variant**	**V72A**					
**Soaking condition**	**aerobic Mn+Fe**					
Crystal	1		2		3	
Beamline	X06SA/SLS	X06SA/SLS	X06SA/SLS	X06SA/SLS	X06SA/SLS	X06SA/SLS
Detector	Pilatus 6MF	Pilatus 6MF	Pilatus 6MF	Pilatus 6MF	Pilatus 6MF	Pilatus 6MF
Dataset	Fe peak	Mn peak	Fe peak	Mn peak	Fe peak	Mn peak
Wavelength (Å)	1.73	1.88	1.73	1.88	1.73	1.88
Resolution range (Å)	50.00-2.99 (3.17-2.99)	50.00-2.99 (3.17-2.99)	50.00-2.99 (3.17-2.99)	50.00-2.98 (3.16-2.98)	50.00-2.97 (3.15-2.97)	50.00-2.99 (3.17-2.99)
Space group	C2	C2	C2	C2	C2	C2
Unit cell dimensions a, b, c (Å)	162.73, 55.79, 69.80	162.58, 55.80, 69.73	162.65, 55.80, 69.72	162.62, 55.83, 69.74	162.63, 55.76, 69.57	162.49, 55.77, 69.46
β (°)	114.23	114.15	114.05	114.07	114.19	114.14
Unique reflections	21831 (3375)	21538 (3278)	21962 (3370)	21885 (3207)	21528 (3045)	21797 (3332)
Multiplicity	3.4	3.3	3.4	3.3	3.3	3.4
Completeness (%)	96.1 (91.9)	94.6 (88.9)	96.4 (91.3)	95.2 (86.4)	93.4 (81.7)	96.7 (92.2)
I/σ(I)	17.11 (6.32)	11.80 (3.82)	12.41 (3.53)	9.41 (2.70)	11.13 (2.93)	7.21 (1.91)
R_{merge} (%)	5.3 (15.9)	7.6 (24.4)	7.8 (29.8)	9.8 (33.4)	8.2 (34.4)	13.8 (52.2)
R_{meas} (%)	6.3 (19.1)	9.1 (29.4)	9.2 (35.8)	11.7 (40.3)	9.9 (41.5)	16.5 (62.8)
$CC_{1/2}$ [a]	99.8 (97.6)	99.6 (93.9)	99.6 (92.6)	99.3 (87.7)	99.5 (89.9)	98.8 (70.1)
Anomalous signal	1.06 (0.81)	0.85 (0.76)	0.98 (0.80)	0.88 (0.77)	0.89 (0.73)	0.83 (0.78)

Values in parentheses are for the highest resolution shell. For native datasets, Friedel pairs were merged [a]Percentage of correlation between intensities from random half-datasets [1]. The correlation is significant at the 0.1% level in all resolution shells in all datasets.

Table S1 (continued) Crystallographic data statistics

**R2lox variant**	**V72I**					
**Soaking condition**	**anoxic Mn+Fe**					
Crystal	1		2		3	
Beamline	X06SA/SLS	X06SA/SLS	X06SA/SLS	X06SA/SLS	X06SA/SLS	X06SA/SLS
Detector	Eiger 16M	Eiger 16M	Eiger 16M	Eiger 16M	Eiger 16M	Eiger 16M
Dataset	Fe peak	Mn peak	Fe peak	Mn peak	Fe peak	Mn peak
Wavelength (Å)	1.73	1.88	1.73	1.88	1.73	1.88
Resolution range (Å)	50.00-3.00 (3.19-3.00)	50.00-3.00 (3.18-3.00)	50.00-2.99 (3.18-2.99)	50.00-3.00 (3.18-3.00)	50.00-3.00 (3.18-3.00)	50.00-2.99 (3.17-2.99)
Space group	I222	I222	I222	I222	I222	I222
Unit cell dimensions a, b, c (Å)	55.91, 98.74, 128.53	55.89, 98.48, 128.41	55.94, 98.59, 128.28	55.97, 98.55, 128.37	55.92, 98.65, 128.20	55.96, 98.77, 128.34
Unique reflections	13654 (2219)	13683 (2166)	13723 (2174)	13707 (2167)	13683 (2175)	13942 (2240)
Multiplicity	6.6	6.7	6.9	6.8	6.9	6.8
Completeness (%)	99.4 (99.2)	99.2 (96.8)	99.3 (97.0)	99.3 (97.0)	99.1 (97.1)	98.1 (91.5)
I/σ(I)	12.91 (7.36)	11.86 (3.13)	18.81 (6.17)	10.97 (2.49)	16.47 (5.02)	12.55 (2.55)
R_{merge} (%)	11.9 (29.7)	11.3 (46.4)	7.6 (26.7)	12.0 (56.6)	8.7 (33.9)	11.9 (56.8)
R_{meas} (%)	12.9 (32.4)	12.2 (50.4)	8.2 (29.0)	13.0 (61.6)	9.4 (36.7)	12.9 (61.6)
$CC_{1/2}$ [a]	99.5 (93.4)	99.7 (87.9)	99.8 (94.0)	99.7 (82.5)	99.8 (90.6)	99.8 (79.6)
Anomalous signal	1.40 (1.67)	0.90 (0.84)	1.17 (0.83)	0.88 (0.78)	1.12 (0.81)	0.89 (0.73)

Values in parentheses are for the highest resolution shell. For native datasets, Friedel pairs were merged [a]Percentage of correlation between intensities from random half-datasets [1]. The correlation is significant at the 0.1% level in all resolution shells in all datasets.

Table S1 (continued) Crystallographic data statistics

**R2lox variant**	**V72I**					
**Soaking condition**	**aerobic Mn+Fe**					
Crystal	1		2		3	
Beamline	X06SA/SLS	X06SA/SLS	X06SA/SLS	X06SA/SLS	X06SA/SLS	X06SA/SLS
Detector	Eiger 16M	Eiger 16M	Eiger 16M	Eiger 16M	Eiger 16M	Eiger 16M
Dataset	Fe peak	Mn peak	Fe peak	Mn peak	Fe peak	Mn peak
Wavelength (Å)	1.73	1.88	1.73	1.88	1.73	1.88
Resolution range (Å)	50.00-2.98 (3.16-2.98)	50.00-2.98 (3.16-2.98)	50.00-3.00 (3.18-3.00)	50.00-2.99 (3.17-2.99)	50.00-3.00 (3.19-3.00)	50.00-2.99 (3.17-2.99)
Space group	I222	I222	I222	I222	I222	I222
Unit cell dimensions a, b, c (Å)	55.56, 95.97, 127.97	55.60, 96.02, 127.98	56.00, 97.40, 129.62	55.95, 97.39, 129.55	56.01, 96.99, 129.75	56.05, 97.05, 129.87
Unique reflections	13278 (2070)	13551 (2172)	13656 (2133)	13676 (2132)	13591 (2160)	13637 (2012)
Multiplicity	6.9	6.7	6.8	6.7	7.0	6.7
Completeness (%)	98.5 (95.3)	97.9 (91.9)	99.1 (95.3)	99.0 (95.1)	99.7 (98.8)	98.3 (90.0)
I/σ(I)	17.87 (6.60)	11.71 (2.77)	23.47 (12.04)	11.88 (2.91)	26.43 (8.88)	8.93 (1.62)
R_{merge} (%)	7.9 (23.8)	11.8 (49.9)	6.0 (14.6)	11.4 (47.6)	5.7 (17.4)	16.8 (83.0)
R_{meas} (%)	8.5 (25.8)	12.8 (54.1)	6.5 (15.8)	12.4 (51.8)	6.2 (18.9)	18.4 (84.4)
$CC_{1/2}$ [a]	99.8 (96.7)	99.6 (88.7)	99.8 (98.7)	99.7 (87.8)	99.9 (98.3)	99.4 (75.8)
Anomalous signal	1.10 (0.78)	0.91 (0.72)	1.38 (1.19)	0.93 (0.78)	1.35 (0.93)	0.88 (0.69)

Values in parentheses are for the highest resolution shell. For native datasets, Friedel pairs were merged [a]Percentage of correlation between intensities from random half-datasets [1]. The correlation is significant at the 0.1% level in all resolution shells in all datasets.

Table S1 (continued) Crystallographic data statistics

**R2lox variant**	**V72L**							
**Soaking condition**	**anoxic Mn+Fe**				**aerobic Mn+Fe**			
Crystal	1		2		1		2	
Beamline	X06SA/SLS	X06SA/SLS	X06SA/SLS	X06SA/SLS	X06SA/SLS	X06SA/SLS	X06SA/SLS	X06SA/SLS
Detector	Pilatus 6MF	Pilatus 6MF	Pilatus 6MF	Pilatus 6MF	Pilatus 6MF	Pilatus 6MF	Pilatus 6MF	Pilatus 6MF
Dataset	Fe peak	Mn peak	Fe peak	Mn peak	Fe peak	Mn peak	Fe peak	Mn peak
Wavelength (Å)	1.73	1.88	1.73	1.88	1.73	1.88	1.73	1.88
Resolution range (Å)	50.00-2.99 (3.17-2.99)	50.00-2.99 (3.17-2.99)	50.00-2.99 (3.17-2.99)	50.00-2.99 (3.18-2.99)	50.00-2.99 (3.17-2.99)	50.00-2.98 (3.16-2.98)	50.00-2.99 (3.17-2.99)	50.00-2.96 (3.13-2.96)
Space group	C2	C2	C2	C2	C2	C2	C2	C2
Unit cell dimensions a, b, c (Å)	160.43, 55.70, 70.00	160.50, 55.72, 70.01	160.59, 55.70, 70.03	160.61, 55.72, 70.05	163.43, 55.74, 69.41	163.34, 55.78, 69.44	163.51, 55.58, 67.20	163.54, 55.59, 67.18
β (°)	114.00	114.01	114.01	114.03	114.08	114.05	113.71	113.71
Unique reflections	21618 (3268)	21512 (3299)	21337 (3266)	21204 (3206)	21739 (3361)	21549 (3194)	20579 (3120)	20512 (2626)
Multiplicity	3.3	3.3	3.5	3.5	3.3	3.2	3.6	3.4
Completeness (%)	95.7 (89.9)	96.0 (91.4)	95.0 (90.6)	94.6 (88.7)	96.0 (92.2)	94.3 (86.4)	93.6 (88.3)	90.0 (71.5)
I/σ(I)	15.03 (8.15)	9.65 (4.85)	17.34 (10.08)	13.08 (7.23)	12.33 (4.99)	9.17 (3.20)	16.28 (7.38)	10.38 (3.20)
R_{merge} (%)	6.4 (13.4)	9.8 (22.9)	5.9 (10.9)	7.8 (15.0)	7.3 (19.9)	9.3 (27.7)	5.7 (14.2)	8.9 (29.9)
R_{meas} (%)	7.6 (16.1)	11.7 (27.8)	7.0 (13.0)	9.1 (17.8)	8.8 (24.1)	11.2 (33.9)	6.7 (16.8)	10.6 (35.7)
$CC_{1/2}$ [a]	99.4 (97.8)	98.9 (93.5)	99.5 (98.4)	99.3 (97.0)	99.5 (95.6)	99.1 (91.5)	99.7 (97.8)	99.4 (90.3)
Anomalous signal	1.08 (1.00)	0.90 (0.98)	1.12 (0.98)	0.82 (0.82)	0.91 (0.76)	0.81 (0.75)	1.08 (0.90)	0.92 (0.87)

Values in parentheses are for the highest resolution shell. For native datasets, Friedel pairs were merged [a]Percentage of correlation between intensities from random half-datasets [1]. The correlation is significant at the 0.1% level in all resolution shells in all datasets.

Table S1 (continued) Crystallographic data statistics

**R2lox variant**	**Y162F**				
**Soaking condition**	**anoxic Mn+Fe**				
Crystal	1	1		2	
Beamline	X06SA/SLS	X06SA/SLS	X06SA/SLS	X06SA/SLS	X06SA/SLS
Detector	Pilatus 6MF	Pilatus 6MF	Pilatus 6MF	Pilatus 6MF	Pilatus 6MF
Dataset	native	Fe peak	Mn peak	Fe peak	Mn peak
Wavelength (Å)	0.98	1.73	1.88	1.73	1.88
Resolution range (Å)	50.00-1.39 (1.48-1.39)	50.00-3.00 (3.18-3.00)	50.00-2.98 (3.16-2.98)	50.00-3.00 (3.18-3.00)	50.00-3.00 (3.18-3.00)
Space group	C2	C2	C2	C2	C2
Unit cell dimensions a, b, c (Å)	161.30, 55.60, 69.97	161.49, 55.66, 70.05	161.51, 55.66, 70.06	161.43, 55.62, 70.00	161.61, 55.62, 70.04
β (°)	113.97	114.02	114.03	114.01	114.09
Unique reflections	108553 (15299)	21896 (3470)	21855 (3127)	21742 (3452)	21694 (3347)
Multiplicity	3.3	3.4	3.4	3.5	3.5
Completeness (%)	95.5 (83.7)	97.4 (95.2)	95.5 (85.0)	96.8 (94.6)	96.5 (91.6)
I/σ(I)	15.08 (1.06)	23.98 (15.13)	16.86 (9.22)	26.08 (17.59)	24.82 (12.98)
R_{merge} (%)	3.6 (98.6)	4.2 (6.6)	5.8 (10.2)	3.9 (5.4)	3.9 (7.4)
R_{meas} (%)	4.2 (122.3)	4.9 (7.8)	6.9 (12.3)	4.7 (6.5)	4.7 (8.8)
$CC_{1/2}$ [a]	99.9 (56.8)	99.7 (99.3)	99.5 (98.5)	99.7 (99.5)	99.8 (99.2)
Anomalous signal	-	1.43 (1.16)	0.95 (0.89)	1.49 (1.21)	1.00 (0.90)

Values in parentheses are for the highest resolution shell. For native datasets, Friedel pairs were merged [a]Percentage of correlation between intensities from random half-datasets [1]. The correlation is significant at the 0.1% level in all resolution shells in all datasets.

Table S1 (continued) Crystallographic data statistics

**R2lox variant**	**Y162F**						
**Soaking condition**	**aerobic Mn+Fe**						
Crystal	1	1		2		3	
Beamline	X06SA/SLS	X06SA/SLS	X06SA/SLS	X06SA/SLS	X06SA/SLS	X06SA/SLS	X06SA/SLS
Detector	Pilatus 6MF	Pilatus 6MF	Pilatus 6MF	Pilatus 6MF	Pilatus 6MF	Pilatus 6MF	Pilatus 6MF
Dataset	native	Fe peak	Mn peak	Fe peak	Mn peak	Fe peak	Mn peak
Wavelength (Å)	0.98	1.73	1.88	1.73	1.88	1.73	1.88
Resolution range (Å)	50.00-1.90 (2.02-1.90)	50.00-2.98 (3.16-2.98)	50.00-2.98 (3.16-2.98)	50.00-2.99 (3.18-2.99)	50.00-2.99 (3.17-2.99)	50.00-2.97 (3.15-2.97)	50.00-2.97 (3.15-2.97)
Space group	C2	C2	C2	C2	C2	C2	C2
Unit cell dimensions a, b, c (Å)	162.75, 55.73, 69.46	162.97, 55.71, 69.39	162.81, 55.73, 69.38	162.84, 55.69, 69.28	162.88, 55.74, 69.33	162.90, 55.68, 68.95	162.78, 55.69, 68.89
β (°)	113.92	114.01	113.98	114.01	114.00	113.78	113.80
Unique reflections	42300 (5645)	21669 (3207)	21348 (3079)	21647 (3423)	21347 (3248)	21668 (3188)	21697 (3121)
Multiplicity	3.4	3.4	3.3	3.4	3.3	3.3	3.3
Completeness (%)	94.2 (78.6)	94.9 (86.7)	93.2 (83.3)	96.4 (94.8)	94.6 (89.8)	94.8 (86.5)	94.8 (84.4)
I/σ(I)	14.87 (1.59)	18.85 (6.64)	10.31 (2.95)	13.72 (4.04)	8.73 (2.23)	12.32 (3.02)	9.46 (2.39)
R_{merge} (%)	4.3 (69.3)	5.0 (15.9)	9.2 (33.4)	7.0 (26.7)	11.0 (41.4)	7.4 (33.4)	9.1 (38.3)
R_{meas} (%)	5.1 (82.8)	5.9 (19.0)	10.9 (40.1)	8.3 (31.8)	13.1 (49.8)	8.8 (40.3)	10.9 (46.4)
$CC_{1/2}$ [a]	99.9 (67.6)	99.8 (97.4)	99.4 (88.1)	99.7 (93.9)	99.3 (84.0)	99.6 (91.9)	99.5 (86.0)
Anomalous signal	-	1.14 (0.91)	0.91 (0.80)	1.00 (0.79)	0.87 (0.74)	0.98 (0.75)	0.88 (0.76)

Values in parentheses are for the highest resolution shell. For native datasets, Friedel pairs were merged [a]Percentage of correlation between intensities from random half-datasets [1]. The correlation is significant at the 0.1% level in all resolution shells in all datasets.

Table S1 (continued) Crystallographic data statistics

R2lox variant	A171F						
**Soaking condition**	**anoxic Mn+Fe**						
Crystal	1	1		2		3	
Beamline	BL14.1/BESSY	X10SA/SLS	X10SA/SLS	X10SA/SLS	X10SA/SLS	X10SA/SLS	X10SA/SLS
Detector	Pilatus 6M	Pilatus 6MF	Pilatus 6MF	Pilatus 6MF	Pilatus 6MF	Pilatus 6MF	Pilatus 6MF
Dataset	native	Fe peak	Mn peak	Fe peak	Mn peak	Fe peak	Mn peak
Wavelength (Å)	0.92	1.73	1.88	1.73	1.88	1.73	1.88
Resolution range (Å)	50.00-2.01 (2.13-2.01)	50.00-2.99 (3.18-2.99)	50.00-2.99 (3.18-2.99)	50.00-2.98 (3.16-2.98)	50.00-2.99 (3.17-2.99)	50.00-3.00 (3.18-3.00)	50.00-3.00 (3.18-3.00)
Space group	I222	I222	I222	I222	I222	I222	I222
Unit cell dimensions a, b, c (Å)	55.98, 98.07, 127.63	55.87, 97.93, 127.37	55.87, 97.92, 127.41	55.92, 97.99, 127.62	55.87, 97.83, 127.67	55.88, 98.01, 127.55	55.90, 98.06, 127.53
Unique reflections	23799 (3753)	13572 (2150)	13569 (2153)	13753 (2168)	13654 (2173)	13392 (2095)	13387 (2112)
Multiplicity	6.6	6.9	6.7	6.8	6.7	6.9	6.8
Completeness (%)	99.7 (98.7)	99.6 (97.7)	99.5 (97.6)	99.4 (96.5)	99.6 (97.6)	98.6 (95.4)	98.7 (96.1)
I/σ(I)	17.47 (1.07)	25.02 (8.68)	25.15 (7.95)	26.56 (8.52)	31.61 (9.51)	26.61 (7.86)	23.02 (5.37)
R_{merge} (%)	8.1 (166.8)	5.7 (18.6)	6.5 (24.4)	5.4 (19.3)	4.4 (16.7)	5.5 (22.0)	6.6 (31.4)
R_{meas} (%)	8.8 (181.1)	6.2 (20.2)	7.1 (26.6)	5.8 (21.0)	4.8 (18.1)	6.0 (23.8)	7.1 (34.0)
$CC_{1/2}$ [a]	99.9 (43.7)	99.9 (98.5)	99.9 (97.6)	99.9 (98.3)	99.9 (98.7)	99.9 (98.2)	99.9 (96.2)
Anomalous signal	-	1.27 (0.88)	1.00 (1.04)	1.34 (0.85)	1.06 (0.85)	1.39 (0.92)	0.96 (0.83)

Values in parentheses are for the highest resolution shell. For native datasets, Friedel pairs were merged [a]Percentage of correlation between intensities from random half-datasets [1]. The correlation is significant at the 0.1% level in all resolution shells in all datasets.

Table S1 (continued) Crystallographic data statistics

**R2lox variant**	**A171F**							
**Soaking condition**	**anoxic Mn+Fe**							
Crystal	4		5		6		7	
Beamline	X10SA/SLS	X10SA/SLS	X06SA/SLS	X06SA/SLS	X06SA/SLS	X06SA/SLS	X06SA/SLS	X06SA/SLS
Detector	Pilatus 6MF	Pilatus 6MF	Pilatus 6MF	Pilatus 6MF	Pilatus 6MF	Pilatus 6MF	Pilatus 6MF	Pilatus 6MF
Dataset	Fe peak	Mn peak	Fe peak	Mn peak	Fe peak	Mn peak	Fe peak	Mn peak
Wavelength (Å)	1.73	1.88	1.73	1.88	1.73	1.88	1.73	1.88
Resolution range (Å)	50.00-2.99 (3.17-2.99)	50.00-2.99 (3.18-2.99)	50.00-2.99 (3.17-2.99)	50.00-3.00 (3.18-3.00)	50.00-2.99 (3.17-2.99)	50.00-3.00 (3.18-3.00)	50.00-3.00 (3.18-3.00)	50.00-3.00 (3.18-3.00)
Space group	I222	I222	I222	I222	I222	I222	I222	I222
Unit cell dimensions a, b, c (Å)	55.89, 98.07, 127.85	55.87, 98.00, 127.83	55.94, 98.18, 127.10	55.94, 98.18, 127.99	55.98, 98.20, 128.07	55.10, 98.20, 128.12	56.07, 98.27, 128.15	56.07, 98.22, 128.15
Unique reflections	13624 (2140)	13653 (2194)	13611 (2064)	13497 (2166)	13680 (2121)	13686 (2174)	13705 (2206)	13695 (2198)
Multiplicity	6.8	6.8	6.9	6.8	6.9	6.9	6.9	6.4
Completeness (%)	99.3 (96.1)	99.7 (98.3)	98.3 (92.2)	98.6 (96.7)	99.1 (94.9)	99.6 (97.6)	99.8 (99.1)	99.7 (98.7)
$I/\sigma(I)$	20.09 (6.10)	20.43 (5.24)	14.74 (6.79)	15.04 (7.66)	17.97 (6.56)	19.11 (7.02)	24.29 (9.71)	22.52 (9.02)
R_{merge} (%)	7.3 (27.2)	7.4 (32.3)	10.1 (21.8)	9.8 (19.1)	8.2 (24.6)	7.5 (21.6)	5.9 (15.8)	6.4 (16.7)
R_{meas} (%)	7.9 (29.5)	8.0 (35.1)	11.0 (23.6)	10.7 (20.7)	8.9 (26.7)	8.1 (23.5)	6.4 (17.1)	6.9 (18.1)
$CC_{1/2}$ [a]	99.8 (96.9)	99.8 (95.2)	99.5 (97.7)	99.4 (98.0)	99.8 (97.4)	99.8 (98.1)	99.8 (98.8)	99.8 (98.7)
Anomalous signal	1.15 (0.83)	0.92 (0.85)	1.10 (0.81)	0.93 (0.88)	1.17 (0.85)	0.94 (0.80)	1.44 (1.02)	0.96 (0.80)

Values in parentheses are for the highest resolution shell. For native datasets, Friedel pairs were merged [a]Percentage of correlation between intensities from random half-datasets [1]. The correlation is significant at the 0.1% level in all resolution shells in all datasets.

Table S1 (continued) Crystallographic data statistics

**R2lox variant**	**A171F**						
**Soaking condition**	**anoxic Mn+Fe**		**aerobic Mn+Fe**				
Crystal	8		1	1		2	
Beamline	X06SA/SLS	X06SA/SLS	X06SA/SLS	X06SA/SLS	X06SA/SLS	X06SA/SLS	X06SA/SLS
Detector	Pilatus 6MF	Pilatus 6MF	Pilatus 6MF	Pilatus 6MF	Pilatus 6MF	Pilatus 6MF	Pilatus 6MF
Dataset	Fe peak	Mn peak	native	Fe peak	Mn peak	Fe peak	Mn peak
Wavelength (Å)	1.73	1.88	1.00	1.73	1.88	1.73	1.88
Resolution range (Å)	50.00-3.00 (3.18-3.00)	50.00-3.00 (3.18-3.00)	50.00-1.95 (2.07-1.95)	50.00-3.00 (3.18-3.00)	50.00-3.00 (3.18-3.00)	50.00-3.00 (3.18-3.00)	50.00-2.99 (3.17-2.99)
Space group	I222	I222	I222	I222	I222	I222	I222
Unit cell dimensions a, b, c (Å)	55.98, 98.31, 127.80	56.00, 98.33, 127.84	55.97, 97.20, 128.23	55.86, 97.19, 128.08	55.87, 97.20, 128.11	55.95, 97.38, 128.69	55.88, 97.24, 128.56
Unique reflections	13685 (2188)	13690 (2185)	25690 (4075)	13511 (2157)	13517 (2164)	13588 (2121)	13582 (2115)
Multiplicity	6.8	6.7	3.7	6.9	6.9	6.7	6.7
Completeness (%)	99.6 (97.7)	99.6 (97.5)	98.7 (98.2)	99.8 (99.2)	99.9 (99.4)	99.4 (96.4)	99.4 (96.4)
$I/\sigma(I)$	24.07 (10.11)	22.17 (8.92)	15.33 (1.47)	17.54 (7.37)	16.65 (6.50)	20.04 (4.97)	20.08 (4.97)
R_{merge} (%)	6.0 (15.4)	6.4 (17.1)	4.5 (87.7)	8.4 (20.5)	8.9 (23.6)	7.6 (33.8)	7.3 (33.2)
R_{meas} (%)	6.5 (16.7)	7.0 (18.6)	5.2 (103.4)	9.1 (22.3)	9.6 (25.4)	8.3 (36.8)	7.9 (36.1)
$CC_{1/2}$ [a]	99.8 (98.9)	99.8 (98.6)	99.9 (60.4)	99.7 (98.1)	99.7 (97.2)	99.9 (94.3)	99.9 (94.5)
Anomalous signal	1.39 (0.97)	0.95 (0.79)	-	1.05 (0.82)	1.03 (0.81)	1.10 (0.78)	1.06 (0.80)

Values in parentheses are for the highest resolution shell. For native datasets, Friedel pairs were merged [a]Percentage of correlation between intensities from random half-datasets [1]. The correlation is significant at the 0.1% level in all resolution shells in all datasets.

Table S1 (continued) Crystallographic data statistics

**R2lox variant**	**A171F**							
**Soaking condition**	**aerobic Mn+Fe**							
Crystal	3		4		5		6	
Beamline	X06SA/SLS	X06SA/SLS	X06SA/SLS	X06SA/SLS	X06SA/SLS	X06SA/SLS	X06SA/SLS	X06SA/SLS
Detector	Pilatus 6MF	Pilatus 6MF	Pilatus 6MF	Pilatus 6MF	Pilatus 6M	Pilatus 6M	Pilatus 6M	Pilatus 6M
Dataset	Fe peak	Mn peak	Fe peak	Mn peak	Fe peak	Mn peak	Fe peak	Mn peak
Wavelength (Å)	1.73	1.88	1.73	1.88	1.73	1.88	1.73	1.88
Resolution range (Å)	50.00-2.95 (3.13-2.95)	50.00-2.97 (3.15-2.97)	50.00-3.00 (3.19-3.00)	50.00-3.00 (3.18-3.00)	50.00-3.00 (3.19-3.00)	50.00-3.00 (3.18-3.00)	50.00-3.00 (3.18-3.00)	50.00-3.00 (3.18-3.00)
Space group	I222	I222	I222	I222	I222	I222	I222	I222
Unit cell dimensions a, b, c (Å)	55.93, 97.58, 128.74	55.96, 97.40, 128.84	55.73, 97.28, 128.20	55.74, 97.27, 128.22	55.87, 97.50, 128.44	55.88, 97.40, 128.36	55.76, 97.10, 128.11	55.76, 97.10, 128.10
Unique reflections	13769 (1799)	13798 (1996)	13457 (2156)	13449 (2119)	13500 (2138)	13505 (2173)	13499 (2192)	13480 (2151)
Multiplicity	6.7	6.5	6.8	6.7	6.7	6.6	6.8	6.7
Completeness (%)	96.5 (78.5)	98.0 (87.7)	99.9 (99.3)	99.6 (97.6)	99.6 (97.6)	99.3 (95.9)	99.7 (98.2)	99.8 (98.7)
I/σ(I)	14.11 (3.42)	12.14 (2.54)	13.46 (5.77)	10.25 (3.40)	8.61 (2.23)	8.06 (1.85)	9.22 (2.89)	9.50 (2.83)
R_{merge} (%)	10.3 (49.3)	12.3 (68.3)	10.5 (22.5)	13.8 (37.7)	17.0 (62.8)	19.3 (73.5)	15.7 (45.7)	15.5 (48.8)
R_{meas} (%)	11.2 (53.8)	13.3 (74.7)	11.4 (24.5)	15.0 (41.2)	18.4 (68.2)	21.0 (80.1)	17.0 (49.7)	16.8 (53.3)
$CC_{1/2}$ [a]	99.7 (89.7)	99.6 (83.6)	99.4 (96.9)	99.3 (91.1)	99.3 (80.0)	99.1 (75.0)	99.2 (89.1)	99.3 (86.8)
Anomalous signal	0.95 (0.80)	0.91 (0.70)	0.94 (0.75)	0.91 (0.83)	0.85 (0.74)	0.90 (0.74)	0.86 (0.73)	0.85 (0.74)

Values in parentheses are for the highest resolution shell. For native datasets, Friedel pairs were merged [a]Percentage of correlation between intensities from random half-datasets [1]. The correlation is significant at the 0.1% level in all resolution shells in all datasets.

Table S2 Refinement statistics

R2lox variant	Y162F		A171F	
**Soaking condition**	**anoxic Mn+Fe**	**aerobic Mn+Fe**	**anoxic Mn+Fe**	**aerobic Mn+Fe**
Crystal	1	1	1	1
Dataset	native	native	native	native
PDB ID	6F6M	6F6L	6F6B	6F65
Resolution range (Å)	43.52-1.39	43.46-1.90	48.62-2.01	48.50-1.95
Reflections used	108378	42235	23791	25686
R_{work}/R_{free} (%)[a]	14.9/18.7	16.6/20.4	18.1/22.6	17.7/20.7
Coordinate error (Å)	0.19	0.25	0.32	0.26
Non-H atoms	4894	4758	2425	2445
Protein residues[b]	556 (3-250;263-286/3-286)	556 (3-286/3-250;263-286)	286 (2-287)	285 (2-286)
Water molecules	218	111	50	60
Ligand molecules	2	2	0	1
Metal ions	6	6	3	3
rmsd bonds (Å)[c]	0.013	0.017	0.019	0.018
rmsd angles (°)[c]	0.984	1.052	1.402	1.094
Ramachandran favored/allowed/ outliers (%)[d]	97.3/2.5/0.2	96.9/3.1/0.0	97.2/2.8/0.0	96.5/3.5/0.0
Clashscore[d]	2.81	2.39	3.20	2.55
Wilson *B* factor ($Å^2$)	22.1	39.0	44.3	43.4
Average *B* factors ($Å^2$)[e,f]				
all atoms	36.7	52.4	58.4	58.9
protein main and side chains	34.8/38.1	52.0/53.0	58.5	59.0
site 1 metal ion	20.1/21.9	32.4/37.2	38.0	36.7
site 2 metal ion	21.8/23.0	38.5/40.8	37.0	39.4
additional metal ions	54.9/44.9	76.3/83.8	80.6	77.1
ligand	51.4/51.1	54.1/58.8	-	70.9
water	37.4	45	47.3	50.5
Occupancies <1.0 [f]				
site 1 metal ion	-	-	-	-
site 2 metal ion	-	-	-	-
additional metal ions	0.82/0.67	0.75/0.84	-	0.70

[a]R_{free} is calculated from a randomly selected subset of approximately 2000 reflections (corresponding to up to 5% of reflections) excluded from refinement. [b]Residues out of the 302 residue full-length protein included in the final model are given in parentheses. [c]Root-mean-square deviation from ideal geometry. [d]Geometry statistics were calculated with MolProbity [2]. [e]Average *B* factors were calculated with Baverage in the CCP4 suite [3]. [f]Where there are 2 molecules in the asymmetric unit, the values for chain A and chain B are given separately where applicable (A/B).

References

1. Karplus PA, Diederichs K (2012) Linking Crystallographic Model and Data Quality. Science 336:1030–1033. doi: 10.1126/science.1218231
2. Chen VB, Arendall 3rd WB, Headd JJ, et al (2010) MolProbity: all-atom structure validation for macromolecular crystallography. Acta Crystallogr Sect D Biol Crystallogr 66:12–21. doi: 10.1107/S0907444909042073
3. Winn MD, Ballard CC, Cowtan KD, et al (2011) Overview of the CCP4 suite and current developments. Acta Crystallogr Sect D Biol Crystallogr 67:235–242. doi: 10.1107/S0907444910045749
